# Mosquitoes of North-Western Europe as Potential Vectors of Arboviruses: A Review

**DOI:** 10.3390/v11111059

**Published:** 2019-11-14

**Authors:** Jean-Philippe Martinet, Hubert Ferté, Anna-Bella Failloux, Francis Schaffner, Jérôme Depaquit

**Affiliations:** 1 Faculté de Pharmacie, Université de Reims Champagne-Ardenne, ANSES, SFR Cap Santé, EA7510 ESCAPE–USC VECPAR, 51 rue Cognacq-Jay, 51096 Reims CEDEX, France; hubert.ferte@univ-reims.fr (H.F.); jerome.depaquit@univ-reims.fr (J.D.); 2 Arbovirus et Insectes Vecteurs, Département de Virologie, Institut Pasteur, 25-28 rue du docteur Roux, 75015 Paris, France; anna-bella.failloux@pasteur.fr; 3 Laboratoire de Parasitologie, Hôpital Maison-Blanche, CHU de Reims, 45 rue Cognacq-Jay, 51100 Reims, France; 4 National Centre for Vector Entomology, Institute of Parasitology, Vetsuisse Faculty, University of Zurich, Rämistrasse 71, 8006 Zürich, Switzerland; fschaffner.consult@gmail.com; 5 Francis Schaffner Consultancy, Lörracherstrasse 50, 4125 Riehen (Basel-Land), Switzerland

**Keywords:** *Aedes*, *Culex*, *Anopheles*, *Culiseta*, transmission, West Nile, Usutu, dengue, Zika, chikungunya

## Abstract

Background: The intensification of trade and travel is linked to the growing number of imported cases of dengue, chikungunya or Zika viruses into continental Europe and to the expansion of invasive mosquito species such as *Aedes albopictus* and *Aedes japonicus*. Local outbreaks have already occurred in several European countries. Very little information exists on the vector competence of native mosquitoes for arboviruses. As such, the vectorial status of the nine mosquito species largely established in North-Western Europe (*Aedes cinereus* and *Aedes geminus*, *Aedes cantans*, *Aedes punctor*, *Aedes rusticus*, *Anopheles claviger s.s*., *Anopheles plumbeus*, *Coquillettidia richiardii*, *Culex pipiens s.l*., and *Culiseta annulata*) remains mostly unknown. Objectives: To review the vector competence of both invasive and native mosquito populations found in North-Western Europe (i.e., France, Belgium, Germany, United Kingdom, Ireland, The Netherlands, Luxembourg and Switzerland) for dengue, chikungunya, Zika, West Nile and Usutu viruses. Methods: A bibliographical search with research strings addressing mosquito vector competence for considered countries was performed. Results: Out of 6357 results, 119 references were related to the vector competence of mosquitoes in Western Europe. Eight species appear to be competent for at least one virus. Conclusions: *Aedes albopictus* is responsible for the current outbreaks. The spread of *Aedes albopictus* and *Aedes japonicus* increases the risk of the autochthonous transmission of these viruses. Although native species could contribute to their transmission, more studies are still needed to assess that risk.

## 1. Introduction

Emerging or re-emerging disease outbreaks caused by arboviruses are increasing in Europe. Usually confined to tropical or subtropical countries, their risk of occurrence in Europe (especially in the countries of Western Europe) was considered as relatively low. In a context of global change, with an increase in travel, arboviruses can more easily expand outside of their historical range. Over the last two decades, arboviruses such as dengue, Zika or chikungunya achieved incursions in European countries [1,2,3], causing autochthonous human infections [4,5,6]. Susceptible vertebrate hosts and competent vectors can interact, under appropriate environmental and climatic conditions, to cause outbreaks. Knowledge about the vector competence of native species and their distribution, however, remains limited. As such, overwintering thermophilic species are less likely to establish in northern European countries, as the annual isotherm becomes colder. At the present time, *Aedes albopictus* is considered as the vector species in autochthonous outbreaks of dengue and chikungunya in France [5]. While the vector competence of invasive species is widely studied [7,8,9,10,11], only a few studies are available on native species [12]. Competent native mosquitoes might have the potential to contribute to or to cause local outbreaks in addition to the risk related to invasive species [13].

In this work, we investigated the vector competence for dengue virus (DENV), Zika virus (ZIKV), chikungunya virus (CHIKV), West Nile virus (WNV) and Usutu virus (USUV) according to the geographical distribution of mosquitoes in eight Western European countries (France-Corsica excluded, Belgium, Germany, United Kingdom, Ireland, The Netherlands, Luxembourg and Switzerland).

## 2. Materials and Methods

Our area of interest is defined as the “Cfb” climate of the Köppen-Geiger climate classification [14]. Cfb defines a temperate oceanic climate (C) without a dry season (f) but with a warm summer (b) [15]. Mainland France was investigated although the south of France is characterized as a “Csa” (temperate with a dry and hot summer) or “Csb” (temperate with a dry and warm summer) category. The island of Corsica categorized as “Csb” was excluded from our analysis [15]. Mosquito inventories were then selected in our area of interest. The last European global inventory compiled by Schaffner et al. in 2001 [16] was used, completed with more recent national sources and finally compared with the last review available [17]. For invasive species, updated European Center for Disease Prevention and Control (ECDC) maps were used (https://www.ecdc.europa.eu/en/disease-vectors/surveillance-and-disease-data/mosquito-maps). The vectorial status of mosquitoes was inferred from experimental data using infections in laboratory and/or viral detections in field-collected populations.

**Figure 1:**
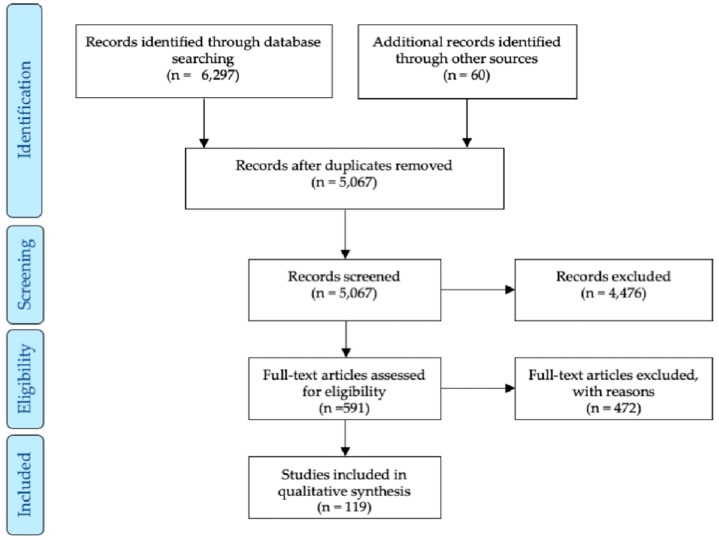
PRISMA flow chart.

This review follows the PRISMA Journal Publishing protocol workflow [18]; the PubMed and Web of Science databases were screened from 11 January to 15 August 2019, with keywords presented on Supplementary File 1. Full-text articles in English language containing information on mosquito vector competence were selected. Articles in other languages containing critical information were included in the data set as “identified by other sources”. The PRISMA flow chart is described in Figure 1.

## 3. Results

The compilation of mosquito inventories is summarized in Table 1. A detailed checklist is shown in Table 2, with the nine ubiquitous species highlighted in grey. Some species complexes (i.e., *Aedes cinereus*/*geminus*, *Anopheles maculipennis* complex, and *Culex pipiens* complex) were not identified at the species and/or biotype level. In these cases, the species denomination was annotated with an asterisk.

Detailed vector competence studies by species are presented in Table 3. Positive and negative results reported here are related to our area of study. Vector competence is defined as the ability of a mosquito to ingest, replicate and transmit a virus by biting. Firstly, species are considered to be competent if viral replication and detection of viral particles in saliva have been demonstrated. On the other hand, we do not consider the detection of viral RNA in mosquito pools as a relevant indication of vector competence. This information, however, may suggest an active virus circulation which could warrant further vector competence studies to identify potential vectors.

Results from experimental infections of European mosquito populations are listed in Table 4.

A Brief Summary of the Reviewed Arboviruses Is Presented Below

### 3.1. Chikungunya Virus

Chikungunya virus (genus *Alphavirus*, family Togaviridae) was reported for the first time in 1952–1953 in Tanzania [66]. The original anthroponotic cycle allows the virus to circulate from *Aedes* mosquitoes to non-human primates. Outbreaks occur when an anthropophilic or opportunistic mosquito (e.g., *Aedes aegypti*, *Ae. albopictus*) completes the bridge between zoonotic and anthroponotic cycles. The girst reported outbreaks occurred in the southern hemisphere during the 1960s [67]. Since then, three lineages have been identified: West-African, East-Central-South-African, and Asian lineages. During the 2000s, a new variant harboring a point mutation at the *E1* glycoprotein gene (E1-A226V) was isolated during the outbreak in the islands of the Indian Ocean, making *Ae. albopictus* more efficient for the transmission of CHIKV [68]. In Europe, the autochthonous transmission of CHIKV was first reported in Italy in 2007 [69]. In our area of interest, the first autochthonous cases of CHIKV occurred in 2010 in Southern France [70]. According to the ECDC, the six countries in this study reported 214 imported cases (Switzerland excluded) in 2012–2016 [71]. An autochthonous circulation of CHIKV was observed in France 2014 and 2017 [4,70,72]. An autochthonous outbreak of CHIKV also occurred in Italy in 2017 [73].

**Figure 2:**
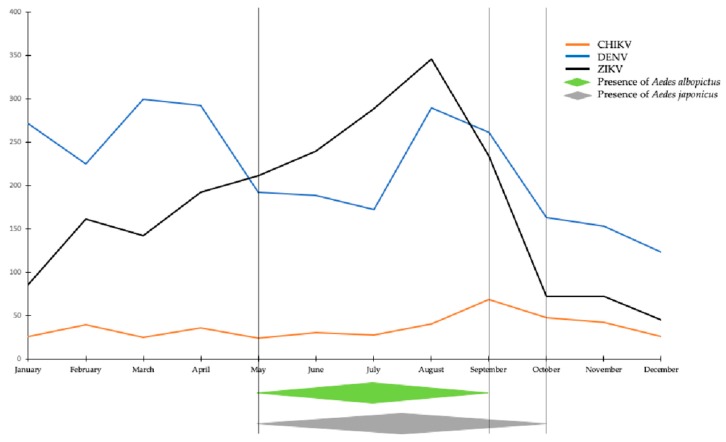
Number of imported cases of chikungunya, dengue and Zika viruses in Europe in 2016 (adapted from [71,74,75]).

French *Ae. albopictus* and German *Ae. japonicus* are competent for CHIKV [11,45]. *Aedes detritus* from the UK was found inefficient to transmit CHIKV [12]. *Aedes vexans* from Northern Italy showed a low disseminated infection rate and its role in CHIKV transmission should not be neglected [13]. In Europe, the declaration of imported cases of CHIKV shows three temporal peaks, namely February, April, and August to November (Figure 2) [71]—periods at which travelers return from endemic areas. While mosquitoes are absent in February, they become active in April; the dynamics of *Ae. japonicus, Ae. koreicus* and *Ae. albopictus* coincide with the peaks of imported cases promoting autochthonous transmission.

### 3.2. Dengue Virus

Dengue virus (genus *Flavivirus*, family Flaviviridae) includes four serotypes (DENV-1 to DENV-4). It is the most widespread arbovirus in the world and is reported in over 100 countries [76]. *Aedes aegypti* and *Ae. albopictus* are the main vectors of DENV acting in urban cycles. According to the latest ECDC annual epidemiological report, covering the 2012–2016 period, 1562 cases (Switzerland excluded) were reported in our study area [74]. The first autochthonous cases of dengue fever were reported in our area of interest (France) in 2010 [77]. Other outbreaks in 2013 and 2015 also occurred in Southern France [5,78], where *Ae. albopictus* is well established and found in abundance [7].

Field-collected *Ae. japonicus* (Germany) and *Ae. detritus* (United Kingdom) were tested for their competence toward a DENV Serotype 2 isolated in Bangkok (Thailand); only *Ae. japonicus* was competent [12,55].

According to the ECDC [74], the frequency of imported cases shows three peaks: January, from March to April, and from August to September (Figure 2). Mosquitoes are only active during the second and third peaks.

### 3.3. Zika Virus

Zika virus (genus *Flavivirus*, family Flaviviridae), was first isolated in the Zika forest of Uganda in 1947 [79]. Since the Yap island epidemic in 2007, ZIKV has circulated on all continents except Europe [80]. There are three main lineages: two from Africa and one from Asia [81]. It is transmitted to humans (urban cycle) and primates (sylvatic cycle) by mosquitoes of the *Aedes* genus. In North-Western Europe, *Ae. albopictus* (France, Germany) and *Ae. japonicus* (Germany) are competent mosquitoes [8,37,46]. Conversely, *Culex pipiens s.l.* and *Cx. pipiens molestus* are not competent for ZIKV [8].

According to the ECDC 2016 annual epidemiological report [75], ZIKV case reports are steadily increasing from January to reach a peak in August then decrease rapidly to end in October (Figure 2). European *Ae. albopictus* is competent to transmit ZIKV [8] and *Ae. japonicus* is poorly competent [37].

### 3.4. Usutu Virus

Usutu virus (genus *Flavivirus*, family Flaviviridae) was initially isolated in South Africa in 1959 [82]. USUV was considered as exclusively transmitted in Africa until the first European outbreak occurred in Italy in 1996 [83]. Since then, USUV emerged in five countries studied in this paper (Belgium, France, Germany, Switzerland, and The Netherlands) [84]. USUV antibodies have been detected in resident and migratory birds in each country of our area of concern [85,86,87,88,89,90], except Ireland and Luxembourg. There are eight lineages of USUV. Five are European (European lineage 1–5) and three are African (African lineage 1–3) [84]. The first human cases were reported in Africa in 1981 [91], and the first human neuroinvasive cases were reported in Italy in 2009 [92]. USUV is transmitted in Europe by *Cx. pipiens* mosquitoes [51]. The reservoir hosts of USUV are migratory and resident birds. USUV or antibodies have been detected in 58 bird species belonging to 26 families and 13 orders [93]. Blackbirds (*Turdus merula*) seem to have the highest mortality rate among bird species affected by USUV [94]. An exhaustive review about WNV and USUV has been published [93]. *Cx. pipiens s.l.* from The Netherlands and the UK have been tested as competent for USUV strains, Bologna 2009 and SAAR-1776 [51,52].

While *Cx. pipiens s.l.* is considered the principal vector in temperate regions, Italian *Ae. albopictus* has been experimentally tested for USUV, with no clear-cut results [47]. *Ae. japonicus* from Austria has been found positive to virus dissemination [95]. To our knowledge, no other mosquito species of NW Europe has been demonstrated to be competent for USUV.

### 3.5. West Nile Virus

West Nile virus (genus *Flavivirus*, family Flaviviridae) was first recorded in the West Nile district of Uganda in 1937 [96]. In natural conditions, it circulates between birds and bird-feeding mosquitoes. Mammals (mainly horses and humans) are considered dead-end hosts [97]. In Europe, WNV is mainly transmitted by *Culex* mosquitoes. The first documented introduction of WNV in metropolitan France dates from 1962, in Camargue [98]. The vector incriminated in 1964 was *Culex modestus* [99]. During the following 40 years, WNV did not cause any human cases in France, and a low prevalence of antibodies was observed in human and equine populations during the 1970s [98]. The resurgence of WNV in France occurred in 2000 with 76 laboratory confirmed equine cases. Sporadic detections of positive serology in humans and birds occurred during the following 15 years [88,100], until a new epidemic outbreak occurred in 2015 in southern France [101]. Three years later, an outbreak occurred for the first time in Germany [102]. The number of cases reported to the ECDC for 2018 was exceptionally high [103].

European mosquitoes display a variable susceptibility to WNV infection and transmission. *Culex spp*. from France, The Netherlands, Switzerland and Germany are competent for WNV lineages 1 and 2. *Culex pipiens* biotype *pipiens*, *Culex pipiens* biotype *molestus* and *Culex torrentium* from the studied area (France, The Netherlands, Switzerland and Germany) are competent for WNV lineage 1 and 2 [41,51,53,55,56,104]. *Culex pipiens s.l.* from Switzerland is susceptible to WNV infection but is not competent for WNV lineage 1 FIN Italy [55]. The competence of *Ae. detritus* (United Kingdom) was demonstrated for WNV lineage 1 strain NY99 [12].

The vector competence of other field-collected species has not been successfully demonstrated for WNV: *Ae. caspius* (France) is susceptible to infection but not able to transmit [41]. *Ae. japonicus* (Germany) could not be infected nor transmit WNV lineage 1 strain NY99 [38].

## 4. Discussion

North-Western Europe is at risk for emerging or re-emerging arboviruses. The epidemiology of arboviruses such as DENV, ZIKV or CHIKV is very different in Europe as compared to tropical or sub-tropical countries. In the latter countries, the existence of sylvatic cycles involving wild animals as reservoir hosts and arboreal canopy dwelling mosquitoes as vectors sustain a viral circulation of viruses all year long [105]. Epidemics only occur when anthropo-zoophilic mosquitoes act as bridge vector for the transmission of the virus from animals to humans. In Europe, in the absence of any sylvatic cycle, autochthonous transmission is only caused when a competent vector becomes infectious after feeding on an imported human case.

The number of imported human cases of CHIKV, DENV and ZIKV peaks in January, March–April, and September–October [71,74,75], corresponding to vacations in Europe. The last two peaks may allow the launch of local transmissions. Regarding the January peak, there is no mosquito activity at that time in the considered area. Consequently, this peak will probably have no consequences regarding a local transmission. During the March–April peak, DENV and CHIKV can be transmitted by *Ae. japonicus* [23], and ZIKV by *Ae. vexans* if European mosquitoes appear to be competent (such as Canadian ones do) [30]. The summer peak is highly correlated with the activity of *Ae. albopictus.* The competence of native populations has been characterized for CHIKV and ZIKV [8,45,46]. Outbreaks have already occurred in southern European countries such as the occurrence of chikungunya in Italy [73] and dengue in France [5]. Established vectors *Ae. albopictus* are also competent for transmitting ZIKV in Italy and Spain [106,107].

To date, *Ae. albopictus* and *Ae. japonicus* are the main species that could represent a risk of the transmission of the considered arboviruses in our area of interest. Indeed, native *Ae. albopictus* and *Ae. japonicus* can transmit CHIKV, DENV, ZIKV [11,37,45,46].

This work highlights that invasive species represent the most probable candidates for the circulation of CHIKV, DENV, and ZIKV in our area of interest. To date, three species are established in our area of interest: *Ae. albopictus*, *Ae*. *koreicus* and *Ae*. *japonicus*. *Ae. albopictus* was first introduced in France in 1999 and has been established since 2004 [49]. It is now widespread and abundant in the southern part of the country. It is also reported as established in the north-eastern part of France, in Germany, in Switzerland, in The Netherlands, and occasionally found in Belgium and the UK [108]. Similarly, *Ae. japonicus* is well established in Belgium, France, Luxembourg, Germany, The Netherlands, and Switzerland [109,110]. *Ae. koreicus* is implanted in Germany, Switzerland, and Belgium [111]. While invasive species benefit from globalization (increased transportation of goods and people) to expand, native species are also experiencing ecological upheavals and contribute to increase the risk of arboviral emergences. Recently, *An. plumbeus* has switched from natural breeding sites to man-made sites [112] leading to consider its potential role as a vector for WNV [36].

While the vector competence of *Ae. albopictus* for the five viruses considered in this study has been largely examined, European native mosquitoes (*Anopheles*, *Culiseta*, *Coquillettidia*, *Uranotaenia*) were poorly investigated. The role of ornithophilic species, such as *Cs. morsitans*, and opportunistic species (mosquitoes that feed on mammalians as well as on birds or amphibians) like *An. plumbeus* or *Ae. geniculatus*, is still unknown, especially in the transmission of USUV and WNV. *An. plumbeus*, however, was tested competent for WNV, and *Ae. geniculatus* for CHIKV and WNV [35,36].

For USUV and WNV, the entanglement of mosquito populations with avian populations is necessary for sustaining the enzootic cycle. Recent years have shown a sharp increase in WNV cases [103]. Although serological and molecular screening is regularly carried out in human [113,114] and avian populations [39,87,115], the screening of mosquito populations is less systematic. Also, WNV antibodies are more prevalent in migratory birds, while USUV are more prevalent in resident birds [115]. USUV was more commonly found in mosquitoes than WNV [115].

Epizootics of WNV are episodic [98]; after the first emergence of WNV in France in 1962 [98], the virus was only detected again in the 2000s [116]. Outbreaks were noted from 2004 to 2018 in mainland France [101,103,117], and in 2018 in Germany [102]. The circulation of USUV was suspected in European birds in 2000–2005 [85,118]. USUV emerged in Germany in 2011 [59], and then in France in 2015 [119]. In 2016, an epizootic has globally affected France, Belgium, Germany and The Netherlands [84]. The species involved in these outbreaks were probably members of the genus *Culex* (e.g., *Cx. pipiens s.l*.) [51,52].

These recent episodes recall our knowledge gaps on the vector competence of native and invasive species such as *Ae. vexans*, *Ae. japonicus* and other *Aedes* species for ZIKV, CHIKV and DENV; *An. plumbeus*, *Ae. geniculatus*, *Cs. annulata*, and *Cx. torrentium* for USUV and WNV. In the future, attention should be given to ubiquitous species which could be of importance if their vector competence happens to be demonstrated.

## Figures and Tables

**Table 1:** Number of mosquito species recorded per country [16,19,20,21,22,23,24,25].

	Belgium	France	Germany	Ireland	Luxembourg	Switzerland	The Netherlands	UK
No. of species	33	57	49	20	15	35	38	34

**Table 2:** Checklist of mosquitoes recorded per country. Species in grey rows were recorded in all the countries investigated in the study. X: species recorded; -: species not recorded; i: introduced species; species complexes for which final identification could not be achieved are labelled with an asterisk.

	Belgium	France	Germany	Ireland	Luxembourg	Netherlands	Switzerland	United Kingdom
*Aedes* (*Acartomyia*) *mariae* (Sergent and Sergent, 1903)	-	X	-	-	-	-	-	-
*Aedes (Aedes) cinereus* Meigen, 1818 * and Aedes (Aedes) geminus Peus, 1970 *	X	X	X	X	X	X	X	X
*Aedes* (*Aedes*) *rossicus* Dolbeskin, Gorickaja and Mitrofanova, 1930	-	X	X	-	-	-	-	-
*Aedes* (*Aedimorphus*) *vexans* (Meigen, 1830)	X	X	X	-	-	X	X	X
*Aedes* (*Dahliana*) *geniculatus* (Olivier, 1791)	X	X	X	-	X	X	X	X
*Aedes (Fredwarsius) vittatus* (Bigot, 1861)	-	X	-	-	-	-	-	-
*Aedes* (*Hulecoeteomyia*) *japonicus* (Theobald 1901)	X	X	X	-	-	X	X	-
*Aedes* (*Hulecoeteomyia*) *koreicus* (Edwards 1917)	X	-	i	-	-	-	X	-
*Aedes* (*Ochlerotatus*) *annulipes* (Meigen, 1830)	X	X	X	-	X	X	X	X
*Aedes* (*Ochlerotatus*) *berlandi* Séguy, 1921	-	X	-	-	-	-	-	-
*Aedes* (*Ochlerotatus*) *cantans* (Meigen, 1818)	X	X	X	X	X	X	X	X
*Aedes* (*Ochlerotatus*) *caspius* (Pallas, 1771)	X	X	X	X	-	X	X	X
*Aedes* (*Ochlerotatus*) *cataphylla* Dyar, 1916	-	X	X	-	-	-	X	-
*Aedes (Ochlerotatus) coluzzi* Rioux, Guilvard and Pasteur, 1998	-	X	-	-	-	-	-	-
*Aedes* (*Ochlerotatus*) *communis* (DeGeer, 1776)	X	X	X	-	-	X	X	X
*Aedes* (*Ochlerotatus*) *detritus* Haliday, 1833	X	X	X	X	-	X	-	X
*Aedes* (*Ochlerotatus*) *diantaeus* Howard, Dyar and Knab, 1912	-	X	X	-	-	-	-	-
*Aedes* (*Ochlerotatus*) *dorsalis* (Meigen, 1830)	X	X	X	X	-	X	X	X
*Aedes* (*Ochlerotatus*) *excrucians* (Walker, 1856)	-	-	X	-	-	X	X	-
*Aedes* (*Ochlerotatus*) *flavescens* (Müller, 1764)	X	X	X	-	-	X	X	X
*Aedes* (*Ochlerotatus*) *leucomelas* (Meigen, 1804)	-	-	X	-	-	X	-	X
*Aedes* (*Ochlerotatus*) *nigrinus* (Eckstein, 1918)	-	X	X	-	-	X	-	-
*Aedes* (*Ochlerotatus*) *pulcritarsis* (Rondani, 1872)	-	X	-	-	-	-	-	-
*Aedes* (*Ochlerotatus*) *pullatus* (Coquillett, 1904)	-	X	X	-	-	-	X	-
*Aedes* (*Ochlerotatus*) *punctor* (Kirby, 1837)	X	X	X	X	X	X	X	X
*Aedes* (*Ochlerotatus*) *riparius* Dyar and Knab, 1907	-	-	X	-	-	X	-	-
*Aedes* (*Ochlerotatus*) *sticticus* (Meigen, 1838)	X	X	X	-	X	X	X	X
*Aedes* (*Ochlerotatus*) *surcoufi* (Theobald, 1912)	-	X	-	-	-	-	-	-
*Aedes* (*Rusticoidus*) *refiki* Medschid, 1928	-	X	X	-	-	-	X	-
*Aedes* (*Rusticoidus*) *rusticus* (Rossi, 1790)	X	X	X	X	X	X	X	X
*Aedes (Stegomyia) aegypti* (Linnaeus,1762)	-	-	-	-	-	i	-	-
*Aedes* (*Stegomyia*) *albopictus* (Skuse, 1894)	X	X	X	-	-	X	X	-
*Anopheles* (*Anopheles*) *algeriensis* (heobald, 1903	-	X	X	X	-	X	-	X
*Anopheles (Anopheles) atroparvus* Van Thiel, 1927	X	X	X	X	-	X	-	X
*Anopheles* (*Anopheles*) *claviger* (Meigen, 1804) *sensu stricto*	X	X	X	X	X	X	X	X
*Anopheles (Anopheles) hyrcanus* (Pallas, 1771)	-	X	-	-	-	-	-	-
*Anopheles* (*Anopheles*) *maculipennis* Meigen, 1818 sensu lato*	X	X	X	-	-	X	X	-
*Anopheles* (*Anopheles*) *melanoon* Hackett, 1934	-	X	-	-	-	X	-	-
*Anopheles* (*Anopheles*) *messeae* Falleroni, 1926	X	X	X	X	-	X	-	X
*Anopheles* (*Anopheles*) *petragnani* Del Vecchio, 1939	-	X	X	-	-	-	-	-
*Anopheles* (*Anopheles*) *plumbeus* Stephens, 1828	X	X	X	X	X	X	X	X
*Coquillettidia* (*Coquillettidia*) *buxtoni* (Edwards, 1923)	-	X	-	-	-	-	X	-
*Coquillettidia* (*Coquillettidia*) *richiardii* (Ficalbi, 1889)	X	X	X	X	X	X	X	X
*Culex* (*Barraudius*) *modestus* Ficalbi, 1889	-	X	X	-	-	X	X	X
*Culex* (*Culex*) *mimeticus* Noé, 1899	-	X	-	-	-	-	-	-
*Culex* (*Culex*) *pipiens* Linnaeus, 1758 sensu lato*	X	X	X	X	X	X	X	X
*Culex* (*Culex*) *pipiens* biotype *pipiens* Linnaeus, 1758	X	-	X	X	-	-	-	X
*Culex* (*Culex*) *pipiens* biotype *molestus* Forskål, 1775	X	-	X	-	-	-	-	X
*Culex* (*Culex*) *theileri* Theobald, 1903	-	X	-	-	-	-	-	-
*Culex* (*Culex*) *torrentium* Martini, 1925	X	X	X	-	X	X	X	X
*Culex* (*Maillotia*) *hortensis* Ficalbi, 1889	X	X	X	-	-	-	X	-
*Culex* (*Neoculex*) *europaeus* Ramos et al., 2003 (syn. *Culex territans* Walker, 1856)	X	X	X	-	X	X	X	X
*Culex* (*Neoculex*) *impudicus* Ficalbi, 1890	-	X	-	-	-	-	-	-
*Culex* (*Neoculex*) *martinii* Medschid, 1930	-	X	X	-	-	-	X	-
*Culiseta* (*Allotheobaldia*) *longiareolata* (Macquart, 1838)	-	X	X	-	-	-	X	X
*Culiseta* (*Culicella*) *fumipennis* (Stephens, 1825)	X	X	-	-	-	X	x	X
*Culiseta* (*Culicella*) *litorea* (Shute, 1928)	-	X	-	X	-	-	-	X
*Culiseta* (*Culicella*) *morsitans* (Theobald, 1901)	X	X	X	X	-	X	X	X
*Culiseta* (*Culicella*) *ochroptera* (Peus, 1935)	-	-	X	-	-	X	-	-
*Culiseta* (*Culiseta*) *alaskaensis* (Ludlow, 1906)	-	X	X	X	-	X	X	X
*Culiseta* (*Culiseta*) *annulata* (Schrank, 1776)	X	X	X	X	X	X	X	X
*Culiseta* (*Culiseta*) *glaphyroptera* (Schiner, 1864)	-	X	X	-	-	-	-	-
*Culiseta* (*Culiseta*) *subochrea* (Edwards, 1921)	X	X	X	X	-	X	-	X
*Orthopodomyia pulcripalpis* (Rondani, 1872)	X	X	-	-	-	-	-	X
*Uranotaenia* (*Pseudoficalbia*) *unguiculata* Edwards, 1913	-	X	X	-	-	-	-	-

**Table 3:** Vector competence studies on European mosquito populations. Negative and positive experiments are written in normal and bold typeface, respectively. Studies related to our area of concern are indicated by a reference number. Studies related to experiments carried out outside of our area of concern are indicated with a reference number and an additional letter: E: Europe; W: elsewhere in the world. Since no capture of chikungunya virus (CHIKV), dengue virus (DENV) and Zika virus (ZIKV) has been reported in our area of concern, these viruses are not mentioned in the field data part of the table.

	Laboratory Experiment					Field Data	
	Dengue	Chikungunya	Zika	Usutu	West Nile	Usutu	West Nile
Aedes cinereus *, Aedes geminus *	-	-	-	-	-	-	[26], W [27]
*Aedes rossicus*	-	-	-	-	-	-	[26], E [28]
*Aedes vexans*	-	**E [13]**	**W [29,30]**	-	**W [31]**	**E [32]**	[26], **E [32]**, **W [27,33,34]**
*Aedes geniculatus*	-	**E [35]**	-	-	**[36]**	-	[26]
*Aedes japonicus*	**[11]**	**[11]**	**[37]**	-	[10,38]	-	**W [39]**
*Aedes koreicus*	-	**E [9]**	-	-		-	-
*Aedes annulipes*	-	-	-	-	-	-	[26]
*Aedes cantans*	-	-	-	-	-	-	**E [40]**
*Aedes caspius*	-	-	-	-	[41]	-	**E [42]**
*Aedes detritus*	[12]	[12]	-	-	**[12]**	**E [42]**	-
*Aedes dorsalis*	-	-	-	-	**W [43]**	-	**W [34]**
*Aedes sticticus*	-	-	W [30]	-	-	-	[26], **W [34]**
*Aedes albopictus*	**W [44]**	**[45]**	**[8,46]**	**E [47]**	**W [48]**	**E [42]**	**W [49]**
*Anopheles claviger sensu lato*	-	-	-	-	-	-	[26]
*Anopheles maculipennis sensu lato* *	-	E [13]	-	-	-	**E [42]**	[26]
*Anopheles plumbeus*	-	-	-	-	**[36]**	-	[26]
*Coquillettidia richiardii*	-	-	-	-	-	-	[26], **E [40]**
*Culex modestus*	-	-	-	-	**[41,50]**	-	[26], E [42]
*Culex pipiens sensu lato* *	-	-	[8]	**[51,52]**	**[41,51,53,54,55,56]**	**[6,57,58,59,60]**, **E [42]**	[26,61], E [42]
*Culex torrentium*	-	-	[8], E [8,62]	-	**[56]**	**[59]**	-
*Culex europaeus*	-	-	-	-	-	**E [32]**	**W [34]**
*Culiseta annulata*	-	-	-	-	-	**E [32,42]**	[26]
*Culiseta morsitans*	-	-	-	-	-	-	**W [40]**
*Uranotaenia unguiculata*	-	-	-	-	-	-	**E [63,64,65]**

*: species complexes for which final identification could not be achieved.

**Table 4:** Experimental infections performed with North-Western European mosquito populations.

Species	Country	Locality	Titer of Blood Meal	Virus Strain	Infection	Transmission	Days Post Infection	Reference
*Aedes vexans*	France	Côte d’Azur	10^7^ PFU/mL	CHIKV 06.21 La Réunion 2005	NA	-	12–14	[7]
*Aedes japonicus japonicus*	Switzerland	Zürich	10^7^ ffu/mL	CHIKV 06.21 La Réunion 2005	+	+	14	[11]
*Aedes japonicus japonicus*	Switzerland	Zürich	10^7^ ffu/mL	DENV Serotype 2, Bangkok Thailand	+	+	14	[11]
*Aedes japonicus japonicus*	Switzerland	Zürich	10^6^ TCID50/mL	West Nile virus (WNV) lineage 1 FIN Italy	+	+	14	[10]
*Aedes japonicus japonicus*	Germany	Stuttgart	2 × 10^7^ PFU/mL	WNV lineage 1 strain NY99	-	-	14	[38]
*Aedes japonicus japonicus*	Switzerland	Zürich	10^6^ TCID50/mL	WNV lineage 1 strain NY99	+	+	14	[10]
*Aedes japonicus japonicus*	Germany	-	10^7^ PFU/mL	ZIKV_FB-GWUH-2016	+	+	14	[37]
*Aedes caspius*	France	Côte d’Azur	10^7^ PFU/mL	CHIKV 06.21 La Réunion 2005	NA	+	12–14	[7]
*Aedes caspius*	France	Camargue	10^10,3^ PFU/mL	WNV PaAn001 AY268135	+	-	14	[41]
*Aedes detritus*	France	Côte d’Azur	10^7^ PFU/mL	CHIKV 06.21 La Réunion 2005	NA	+	12–14	[7]
*Aedes detritus*	United Kingdom	Little Neston	10^7^ PFU/mL	CHIKV NC/ 2011-568	-	-	17	[12]
*Aedes detritus*	United Kingdom	Little Neston	10^7^ PFU/mL	DENV Serotype 2, Bangkok Thailand	-	-	17	[12]
*Aedes detritus*	United Kingdom	Little Neston	2 × 10^6^ PFU/mL	WNV NY-99	+	+	17	[12]
*Aedes albopictus*	France	Bar-sur-Loup	10^6,5^ PFU/mL	CHIKV 06.21 La Réunion 2005	+	+	3,5,7	[45]
*Aedes albopictus*	France	Côte d’Azur	10^7^ PFU/mL	CHIKV 06.21 La Réunion 2005	NA	+	12–14	[7]
*Aedes albopictus*	France	Bar-sur-Loup	10^6,5^ PFU/mL	CHIKV 20235 2013	+	+	3,5,7	[45]
*Aedes albopictus*	France	Nice	10^7^ TCID50/mL	ZIKV strain (NC-2014-5132)	+	+	14	[46]
*Aedes albopictus*	Germany	-	10^7^ PFU/mL	ZIKV_FB-GWUH-2016	+	+	21	[8]
*Culex modestus*	France	Camargue	10^10,3^ PFU/mL	WNV PaAn001 AY268132	+	+	14	[50]
*Culex modestus*	France	Camargue	10^10,3^ PFU/mL	WNV PaAn001 AY268134	+	+	14	[41]
*Culex pipiens* hybrid form	United Kingdom	-	10^6^ PFU/mL	Usutu virus (USUV) African strain SAAR-1776	-	-	14	[52]
*Culex pipiens* hybrid form	Netherlands	-	5.2 × 10^7^ TCID50 /mL	WNV lineage 2 strain Greece 2010	+	+	14	[54]
*Culex pipiens molestus*	Germany	Heidelberg, Wendland, and Langenhelsten	1–1.6 × 10^7^ PFU/mL	WNV lineage 1 strain NY99	+	+	14	[56]
*Culex pipiens molestus*	Netherlands	Amsterdam	5.2 × 10^7^ TCID50 /mL	WNV lineage 2 strain Greece 2010	+	+	14	[54]
*Culex pipiens molestus*	Germany	Langenhelsten	10^7^ PFU/mL	ZIKV_FB-GWUH-2016	+	-	-	[8]
*Culex pipiens pipiens*	United Kingdom	-	10^6^ PFU/mL	USUV African strain SAAR-1776	+	+	14	[52]
*Culex pipiens pipiens*	Germany	Hamburg	1–1.6 × 10^7^ PFU/mL	WNV lineage 1 strain NY99	+	+	14	[56]
*Culex pipiens pipiens*	Netherlands	Best	5.2 × 10^7^ TCID50 /mL	WNV lineage 2 strain Greece 2010	+	+	14	[54]
*Culex pipiens pipiens*	Germany	-	10^7^ PFU/mL	ZIKV_FB-GWUH-2016	+	-	-	[8]
*Culex pipiens s.l.*	France	Côte d’Azur	10^7^ PFU/mL	CHIKV 06.21 La Réunion 2005	NA	-	12–14	[7]
*Culex pipiens s.l.*	Netherlands	Brummen	4 × 10^7^ TCID50/mL	USUV Bologna 2009	+	+	14	[51]
*Culex pipiens s.l.*	Switzerland	Zürich	10^2.6^ to 10^4.2^ PFU/mL	WNV lineage 1 FIN Italy	+	-	14	[55]
*Culex pipiens s.l.*	Switzerland	Zürich	10^2.6^ to 10^4.2^ PFU/mL	WNV lineage 1 strain NY99	+	+	14	[55]
*Culex pipiens s.l.*	Netherlands	Brummen	1.4 × 10^8^ TCID50/ml	WNV lineage 2 strain Greece 2010	+	+	14	[53]
*Culex pipiens s.l.*	Netherlands	Brummen	4 × 10^7^ TCID50/mL	WNV lineage 2 strain Greece 2010	+	+	14	[51]
*Culex pipiens s.l.*	France	Camargue	10^10,3^ PFU/mL	WNV PaAn001 AY268133	+	+	14	[41]
*Culex torrentium*	Germany	Hamburg	1–1,6 × 10^7^ PFU/mL	WNV lineage 1 strain NY99	+	+	14	[56]
*Culex torrentium*	Germany	-	10^7^ PFU/mL	ZIKV_FB-GWUH-2016	+	-	14,21	[8]

## Supplementary Materials

The following are available online at https://www.mdpi.com/1999-4915/11/11/1059/s1, Document S1: Search terms.
