# Supplementary material for: Mosquitoes of North-Western Europe as Potential Vectors of Arboviruses: A Review
Source: Viruses. 2019 Nov 14;11(11):1059. doi: 10.3390/v11111059 (PMC6893686; doi:10.3390/v11111059)
Supplement: Supplementary file 1 [file viruses-11-01059-s001.pdf]

Call for inventories:

ALL= ((inventory OR checklist OR diversity) AND (mosquitoes OR culicidae OR mosquito) AND (Belgium OR France OR Germany OR Ireland OR Luxembourg OR Netherlands OR Switzerland OR United Kingdom))

For vectoral competence of native species and wild carriage:

ALL= ((Usutu OR (West AND Nile) OR dengue OR chikungunya OR Zika) AND (virus OR arbovirus) AND (Belgium OR France OR Germany OR Ireland OR Luxembourg OR Netherlands OR Switzerland OR United Kingdom) AND (vector OR competence OR mosquito OR mosquitoes OR culicidae))

Call for vector competence or wild carriage:

ALL= ((Usutu OR (West AND Nile) OR dengue OR chikungunya OR Zika) AND (virus OR arbovirus) AND (Belgium OR France OR Germany OR Ireland OR Luxembourg OR Netherlands OR Switzerland OR United Kingdom) AND (vector OR competence OR mosquito OR mosquitoes OR culicidae))
